# Astrocytes mediate cerebral blood flow and neuronal response to cocaine in prefrontal cortex

**DOI:** 10.21203/rs.3.rs-2626090/v1

**Published:** 2023-03-20

**Authors:** Yingtian Pan, Congwu Du, Kicheon Park, Yueming Hua, Nora Volkow

**Affiliations:** Stony Brook University; Stony Brook University; National Institute on Drug Abuse National Institutes of Health

**Keywords:** Optical coherence Doppler tomography, Neuro-glio-vascular network, Calcium fluorescence imaging, Cerebral blood flow, Chemogenetics, Prefrontal cortex, Cocaine

## Abstract

Cocaine affects both cerebral blood vessels and neuronal activity in brain. Cocaine can also disrupt astrocytes, which are involved in neurovascular coupling process that modulates cerebral hemodynamics in response to neuronal activity. However, separating neuronal and astrocytic effects from cocaine’s direct vasoactive effects is challenging, partially due to limitations of neuroimaging techniques to differentiate vascular from neuronal and glial effects at high temporal and spatial resolutions. Here, we used a newly-developed multi-channel fluorescence and optical coherence Doppler microscope (fl-ODM) that allows for simultaneous measurements of neuronal and astrocytic activities alongside their vascular interactions *in vivo* to address this challenge. Using green and red genetically-encoded Ca^2+^ indicators differentially expressed in astrocytes and neurons, fl-ODM enabled concomitant imaging of large-scale astrocytic and neuronal Ca^2+^ fluorescence and 3D cerebral blood flow velocity (CBFv) in vascular networks in the mouse cortex. We assessed cocaine’s effects in the prefrontal cortex (PFC) and found that the CBFv changes triggered by cocaine were temporally correlated with astrocytic Ca^2 +^_A_ activity. Chemogenetic inhibition of astrocytes during the baseline state resulted in blood vessel dilation and CBFv increases but did not affect neuronal activity, suggesting modulation of spontaneous blood vessel’s vascular tone by astrocytes. Chemogenetic inhibition of astrocytes during cocaine challenge prevented its vasoconstricting effects alongside the CBFv decreases but also attenuated the neuronal Ca^2+^_N_ increases triggered by cocaine. These results document a role of astrocytes both in regulating vascular tone of blood flow at baseline and for mediating the vasoconstricting responses to cocaine as well as its neuronal activation in the PFC. Strategies to inhibit astrocytic activity could offer promise for ameliorating vascular and neuronal toxicity from cocaine misuse.

## Introduction

Cocaine directly affects both cerebral vessels and neuronal activity in the brain. Glia, in particular, astrocytes, are involved in neurovascular coupling (NVC) – a process that modulates cerebral hemodynamics in response to changes in neuronal activity, which is disrupted by cocaine^1^. Astrocytes interact with neurons and the surrounding blood vessels through their endfeet and processes that ensheath blood vessels and synapses^2^. Astrocyte processes terminate the action of glutamate released by neurons via the glutamate-glutamine cycle^3^ and mediate CBF by modulating NVC^3^. Clinical studies have reported neuronal, astrocytic and vascular pathological alterations in the brain of cocaine users, including neuronal loss, reduction of glial fibrillary acidic protein (GFAP)-immunopositive astrocytes and reactive and degenerative changes of the cerebral micro vasculature^4^. Astrocytes have also been shown to restore synaptic glutamate homeostasis in the NAcore after repeated cocaine exposure in rodents, and their manipulation can attenuate relapse after cocaine withdrawal^5^ indicative of cocaine’s affects in neuro-glio-vascular (NGV) processes.

Cocaine’s effects on cerebral blood vessels versus those in neurons and astrocytes are confounded by their interactions and change with chronicity of cocaine exposures. Thus, distinguishing these effects ideally requires simultaneous multi-parameter measurements performed longitudinally *in vivo*. Moreover, studying the role of astrocytes in brain function is difficult because their removal causes neuronal death^3^. Thus, much of what we know about astrocyte function has resulted from studies of isolated mammalian astrocytes *in vitro*, which cannot inform on how they interact with neurons and the surrounding vessels. Additionally astrocytes are hard to study with electrophysiological tools due to their slow changes in membrane potentials^3^. However, since astrocyte activation involves intracellular Ca^2+^ increases, the detection of astrocytic Ca^2+^ (Ca^2 +^_A_) signaling can be used to monitor their activity. Electrophysiological and Ca^2+^ imaging studies using rodent brain slices have led to new insights into astrocyte-neuron interactions and to astrocytes’ role in the activity of neuronal networks^6–8^. Also, with advances in *in vivo* imaging techniques such as two-photon microscopy (TPM), astrocyte activity and its correlation with cerebral blood flow are now monitored in living brains^9^. However, studies on Ca^2 +^_A_ signaling have been mostly conducted at the single-astrocyte level within a small field of view (FOV) accessible such as by TPM rather than over larger astrocyte populations, from which synchronized Ca^2 +^_A_ signaling arises^10,11^. Indeed, the behavior of synchronized Ca^2 +^_A_ signaling from large-scale astrocyte populations (e.g., astrocyte ensembles) is not well understood and neither is their functional role in NGV interactions.

Genetically encoded Ca^2+^ fluorescence indicators (GECIs) such as green GCaMP6f enabled us to image cortical neuronal or astrocytic Ca^2+^ fluorescence signals in separate groups of animals^12, 13^. Recently, jRGECO1a, a sensitive red GECI was used to image neuronal activity^14–16^. Therefore, if combined with a green GECI (e.g., GCaMP6f), both indicators could be used to simultaneously image neuronal and astrocytic activities in the same animal allowing for studying their interactions. To achieve this, we developed a spectrally resolved fluorescence imaging system capable of distinguishing two-color fluorescence emissions.

Advances in optical coherence tomography (OCT) for 3D vascular imaging have led to OCT angiography (OCTA or OCA) to visualize the vasculature^17–20^ and optical coherence Doppler tomography (ODT)^20,21^ for quantitative CBF velocity (CBFv) imaging. We reported simultaneous imaging with ultrahigh-resolution OCA (μOCA) and ODT (μODT) based on phase-intensity-multiplexing to concomitantly obtain 3D microangiography and quantitative CBFv measures at capillary resolution^21,22^. 3D μODT measures intrinsic Doppler effect of moving red blood cells to image CBFv circumventing the need for a contrast agent. μODT allows 3D imaging of CBFv in arteries, veins, and capillaries ^23^ with high sensitivity (< 20μm/s) and a large field of view (e.g., 3x2.4x1.4mm^3^). Such an ultrahigh-resolution CBFv imaging technique provides a powerful tool to study the role of astrocytes in NGV interactions and to investigate cocaine’s effect.

Here, we applied a novel multi-channel fluorescence and μOCA/μODT microscope (fl-ODM), which enabled us to concomitantly image large-scale astrocytic and neuronal Ca^2+^ fluorescence and 3D CBFv in vascular networks of the mouse cortex. We used fl-ODM to acquire genetically encoded Ca^2+^ fluorescence images of cellular activities in neurons (Ca^2 +^_N_ with jRGECO1a^14^) and in astrocytes (Ca^2 +^_A_ with GCaMP6f^12^) alongside the CBFv effects in response to cocaine in the prefrontal cortex (PFC) of GFAP-cre mice^12^ *in vivo*. We hypothesized that cocaine enhances Ca^2 +^_A_ accumulation^24^, increasing the vulnerability of the brain to ischemia and thus jeopardizing neuronal activities in the PFC. To assess the role of astrocytes in modulating NGV interactions in responses to cocaine, we used chemogenetics (Designer Receptors Exclusively Activated by Designer Drugs or DREADDs) to inhibit Ca^2 +^_A_ accumulation (e.g., GFAP-DREADDs(Gi)), which we hypothesized would reduce cocaine-induced neuronal Ca^2 +^_N_ activity and ameliorate CBF decrease in PFC.

## Results

### 1. Spectrally-resolved fluorescence and optical coherence Doppler microscopy for simultaneous imaging of neuronal, astrocytic and microvascular dynamics

**Figure 1:**
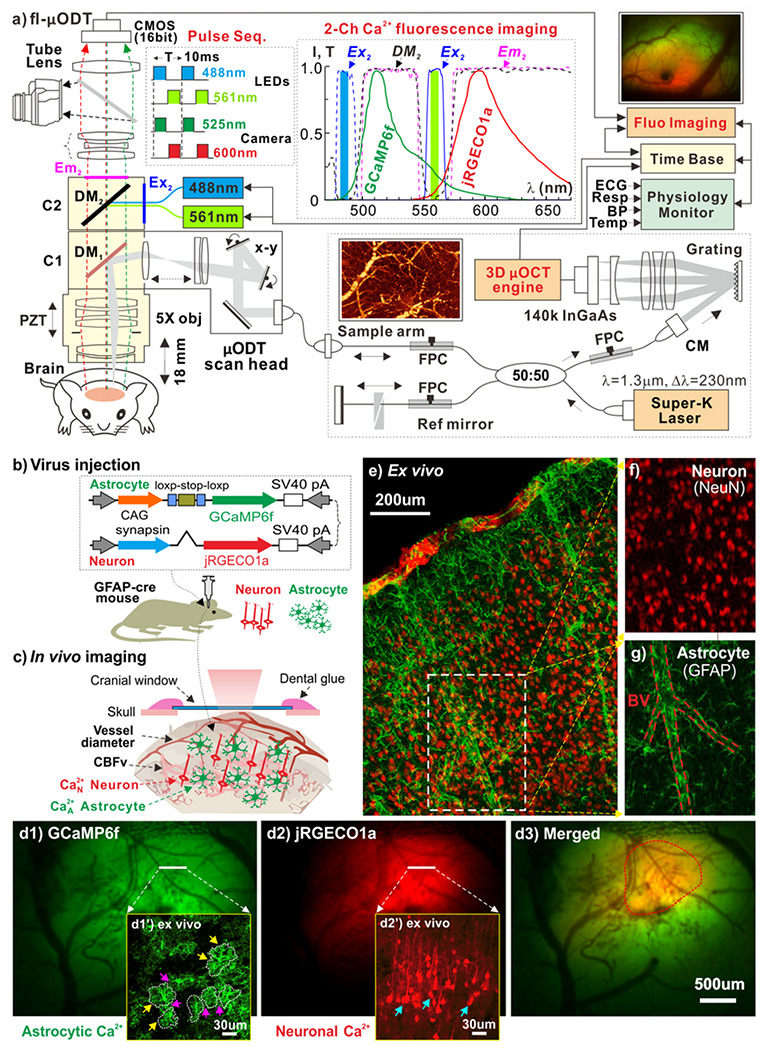
a) Hybrid fl-ODM for simultaneous 3D mOCA/mODT of microvasculature/CBFv (2.4×2×1.2mm^3^) and 2-channel neuronal and astrocytic Ca^2+^ fluorescence imaging (4×3mm^2^, 80fps) of mouse cortex. C1: dichroic mirror (λ_DM_≈1.1μm) to reflect 1.3μm μODM light and transmits GCaMP6f (520nm) and jRGECO1a (600nm) Ca^2+^ fluorescence; C2: spectral-multiplex epi-fluorescence cube to separate green GCaMP6f (λ_EX1_=485±12nm, λ_DM1_=495nm, λ_EM1_ = 520±20nm) and red jRGECO1a (λ_EX2_=559±8nm, λ_DM2_=573nm, λ_EM2_≥574nm) fluorescence emissions to simultaneously image astrocytic and neuronal Ca^2+^ activities by a sCMOS camera (Zyla 5.5, Andor) at up to 80fps. Spectral-domain mOCT engine: illuminated by an ultrabroad-band source (Super-K laser: p>100mW, λ=1.3μm, Δλ=230nm) and detected by fast 2k InGaAs array (GL2048R, Sensors Unlimited) at up to 140kpfs; CM, FPC: fiberoptic collimator, polarization controller; Time base: digital I/O to synchronize 2 image workstations for fluorescence and μOCA/μODT imaging; PZT: piezoelectric actuated focal tracking; Base optics: a modified Nikon FN-1 microscope with a broadband 5x obj (e.g., LSM03, Thorlabs); μODT scan head: a custom 2D confocal laser scanner to interconnect FN1 and μOCT engine, **b)** Viral injection to express Ca^2+^ in astrocytes (Ca^2+^_A_ - GCaMP6f) and neurons (Ca^2+^_N_ - jRGECO1a) in the cortex of GFAP-cre mice, **c)** A sketch to illustrate fl-ODM for simultaneous imaging of neuro-astroglio-vascular interactions *in vivo*. **d1-d3**) *In vivo* images of Ca^2+^_A_ (GCaMP6f) and Ca^2+^_N_ (jRGECO1a) channels and their merged images, where the inserts (**d1’, d2’**) show the *ex vivo* confirmation of cell-specific expressions; **e**) *Ex vivo* confocal image to show the distribution of astrocytes (labeled by GFAR green), neurons (labeled by NeuN, red). GFAP: antibody of fibrillary acidic protein to visualize astrocytes; NeuN: antibody for neuronal nuclei; **f-g**) Neuron and astrocyte distributions in the dashed area in **e**), indicating that astrocytes unsheathe the microvessels (dashed red lines) in the brain to likely mediate microflows.

Figure 1a illustrates a custom hybrid dual-channel fluorescence and ultrahigh-resolution optical coherence Doppler microscope (fl-ODM) which combines the two imaging modalities into an upright microscope body (FN1, Nikon) via epi-fluorescence cube turrets (C1, C2) for *in vivo* small animal studies. An ultrahigh-resolution optical coherence angiography (μOCA) and Doppler tomography (μODT) system in the near infrared range (λ=1.3μm, Δμ=230nm) was integrated through a dichroic mirror (μ_1DM_=1.1μm) in C1 to provide 3D images of the microvasculature and quantitative cerebral blood flow velocity (CBFv) in vascular networks in the mouse cortex over a large field of view (FOV, e.g., 2.4⋅2⋅1.2mm^3^) with capillary resolution (e.g., <5μm). The technical details of 3D μOCA/μODT were previously reported^23^ except a custom high-fidelity 2D confocal laser scanning module to interconnect the μOCT engine to fl-ODM. In parallel, a custom epifluorescence cube (C2) is used for 2-channel spectral-multiplex imaging of the synchronized intracellular calcium fluorescence changes in astrocytes (Ca^2+^_A_ expressed with GCaMP6f: λ_EX1_=485±12nm, λ_DM1_=495nm, λ_EM1_=520±20nm) and in neurons (Ca^2+^_N_ expressed with jRGECO1a: λ_EX2_=559±8nm, λ_DM2_=573nm, λ_EM2_≥574nm) over a larger FOV (e.g., 4⋅3mm^2^). Pulsed high-power narrow-band blue (488nm) and yellowish green (560nm) LEDs from a light engine (Aura III, Lumencor) were used for time-sharing spectral excitation and synchronized with a SCMOS camera (Zyla 5.5, Andor) for sequential fluorescence image acquisition (T=10ms exposure per channel). Ca^2+^_N_(t) and Ca^2+^_A_(t) imaged at up to 80fps were quantified as the relative florescence changes (ΔF/F) vs their baselines to represent neuronal or astrocyte activities, respectively.

To define the mechanisms by which astrocytic and neuronal activities are involved in NGV interactions *in vivo*, we used viral injection to express genetically encoded Ca^2+^ indicators in mice in a cell-specific manner. Figure 1b illustrates our approach to express astrocytic Ca^2+^_A_ and neuronal Ca^2+^_N_ in the PFC *in vivo*. The use of GFAP-cre mouse with delivery of two mixed viral vectors including 50% AAV5.CAG.Flex.GCaMP6f.WPRE.SV40 (#100835, Add-gene) and 50% AAV1.Syn.NES-jRGECO1a.WPRE.SV40 (100854, Add-gene) allowed us to express GCaMP6f for Ca^2+^_A_ fluorescence and jRGECO1a for Ca^2+^_N_ fluorescence in the cortex. Prior to imaging, a cranial window was implanted above the PFC as illustrated in Fig. 1c (see Method Section for details). Figures 1d1–d3 show simultaneous *in vivo* images of Ca^2+^_A_ (**d1**) and Ca^2+^_N_ (**d2**) fluorescence from PFC of a GFAP-cre mouse at ~4wks after viral injection of synapsin jRGECO1a for neurons and cre-GCaMP6f for astrocytes. *Ex vivo* double staining with GFAP antibody to label astrocytes and NeuN antibody to label neurons confirmed astrocyte- (**d1’**) and neuron- (**d2’**) specific Ca^2+^ expressions. Astrocytes also modulate neuro-vascular coupling (NVC) through their process endfeet forming close interactions with neurons and microvessels^3^. Our *ex vivo* images (Figs. 1e–g) show that astrocytes (labeled by GFAP, green) ensheathe neurons (labeled by NeuN, red) and microvessels (red dashed lines – GFAP of astrocyte endfeet) in the mouse cortex (**e, g**).

### 2. Cocaine increased neuronal Ca^2+^_N_ and astrocytic Ca^2+^_A_ activity and decreased CBFv

**Figure 2:**
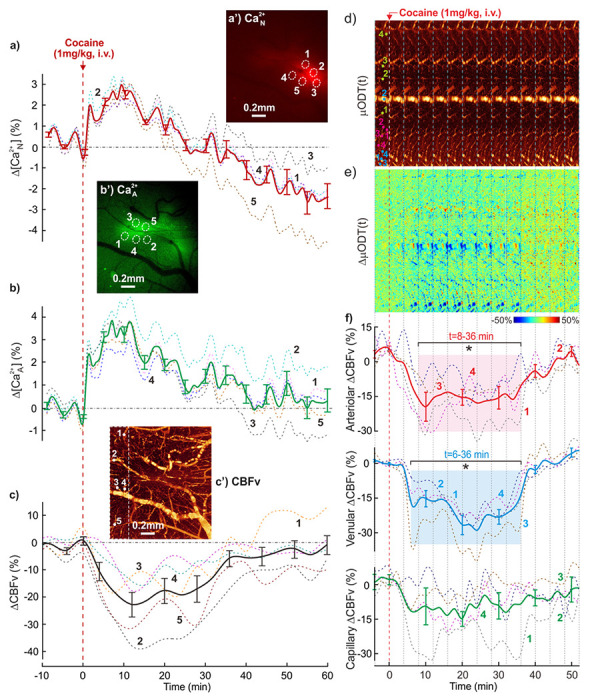
Simultaneous imaging of Ca^2+^_N_ (**a’**) and Ca^2+^_A_ (**b’**) fluorescence along with 3D μODT of the local CBFv network (**c’**) in the PFC of a GFAP-mouse. Five regions of interest (ROIs) from fluorescence-expressing regions of jRGECO1a for Ca^2+^_N_ (circles in **a’**) and GCaMP6f for Ca^2+^_A_ (circles in **b’**) as well as 5 vessels (**c’**) were selected to track their dynamic changes before and after acute cocaine at t=0min (1 mg/kg, i.v.) **a**)-**c**) Time courses of mean Ca^2+^ fluorescence changes in neurons (ΔCa^2+^_N_, solid line, m=5) and astrocytes (ΔCa^2+^_A_, solid line, m=5) along with CBFv network changes in response to cocaine. Increases in ΔCa^2+^_N_ and ΔCa^2+^_A_ indicate activation of neurons and astrocytes by cocaine whereas the decrease in CBFv presumably reflects the vasoconstricting effects of cocaine. Cocaine-induced CBFv network changes in PFC. **d**)-**f**) Time-lapse 3D μODT images of CBFv dynamic responses of all 3 vascular components to cocaine, μODT(t), and their ratio changes ΔμODT(t) over the baseline (t<0min), ΔμODT(t) (image size: 2×0.3×1.2mm^3^); **d**) Cocaine-induced CBFv changes in arteriolar (AF), venular (VF) and capillary (CF) flow networks, in which 4 flows in each compartment were tracked to quantify their mean variations (bold curves) shown in **f**). *: periods showing significant flow decreases (p<0.001, m=4). ROI labels: 1-4, 1-4 and 1-4 refer to arteries, veins and capillaries.

To demonstrate the technical capability of fl-ODM for tracking neuronal, astrocytic and vascular changes in real time, we simultaneously imaged the mouse PFC (A/P: +2.5; M/L: 0.5; D/V: −0.5 mm) to detect activations of Ca^2+^_N_, Ca^2+^_A_ fluorescence and local CBFv changes elicited by an acute cocaine challenge (1 mg/kg, *i.v.*). Figures 2a’–c’ show representative Ca^2+^_N_, Ca^2+^_A_ and CBFv images obtained from the PFC of a GFAP-mouse at baseline (before cocaine). To assess the dynamic changes in Ca^2+^_N_, Ca^2+^_A_ and CBFv from time of cocaine injection, five regions of interest (ROIs, i.e., white circles illustrated in **a’** and **b’**) were selected in the cortex within the fluorescence expressing regions and five in vessels in the surrounding area as shown in Fig. 2c’. Figures 2a–c show the time courses of Ca^2+^_N_, Ca^2+^_A_ and CBFv in response to cocaine, respectively. Cocaine increased Ca^2+^_N_ and Ca^2+^_A_ activities (Figs. 2a–b), whereas it decreased CBFv in cerebrovascular trees (**c**). Specifically, cocaine triggered a Ca^2+^_N_ increase to a maximum of 2.92%±0.20% (m=5) over baseline at t_p–N_=8.6±0.4min, after which it recovered within t_r–N_=27.6±3.3min followed by a downshoot. Similarly, cocaine increased Ca^2+^_A_ to a maximum of 3.87%±0.23% (m=5) at t_p–A_=10.6±0.4min, peaking slightly behind the t_p–N_ of Ca^2+^_N_ (p=0.008, m=5). However, Ca^2+^_A_ did not return to baseline until t_r–A_=50.6±5.6min (m=5), indicating a longer-lasting effect of cocaine on astrocytes than on neurons (p=0.01, m=5). Meanwhile, cocaine decreased CBFv to −22.8%±5.1% (m=5) below baseline at t_p–V_=16.6±2.2min followed by a gradual recovery by t_r–V_=58.6±5.8min, also indicative of a long-lasting CBFv decrease presumably due to vasoconstriction.

In addition, fl-ODM allowed us to concomitantly measure cocaine-induced transient CBFv changes in arteries, veins and capillaries. Figures 2d–e show time-lapse μODT images and the ratio changes over the baseline of a smaller volume (2×0.3×1.2mm^3^, marked by a dished box in Fig. 2c’) in the mouse PFC acquired to quantify dynamic flow changes (2min/volume) from baseline (t<−4min) to t>50min after cocaine (1 mg/kg, *i.v.*, t=0min). The 3D μODT image in Supplemental **Fig.S1** shows that the selected ROIs included pial flows in layer 1 and deep flows in layers 4–5. Under isoflurane anesthesia, all three vessel compartments (**f**) showed flow decreases after cocaine injection, among which arteriolar (AF) and venular(VF) flows dropped −19.8%±6.3% (p<0.001, m=4; t=8-36min) and −29.3%±4.5% (p<0.001, m=4; t=6-36min), respectively, followed by a gradual recovery to their baselines at 49.5±4.6min and 45.5±3.2min, respectively. Although capillary flows (CF) showed an overall decrease to −13.5%±3.0% that peaked at 20±2.9min, individual flow changes varied, with increases over 7% in some capillaries and decreases over −35% in others. The heterogeneity in the capillary responses highlights the importance of measuring flow in multiple capillaries instead of isolated vessels when studying NGV interactions and its responses to cocaine.

### 3. Astrocytic Ca^2+^_A_, but not neuronal Ca^2+^_N_, correlated with cocaine-induced CBFv decreases

**Figure 3:**
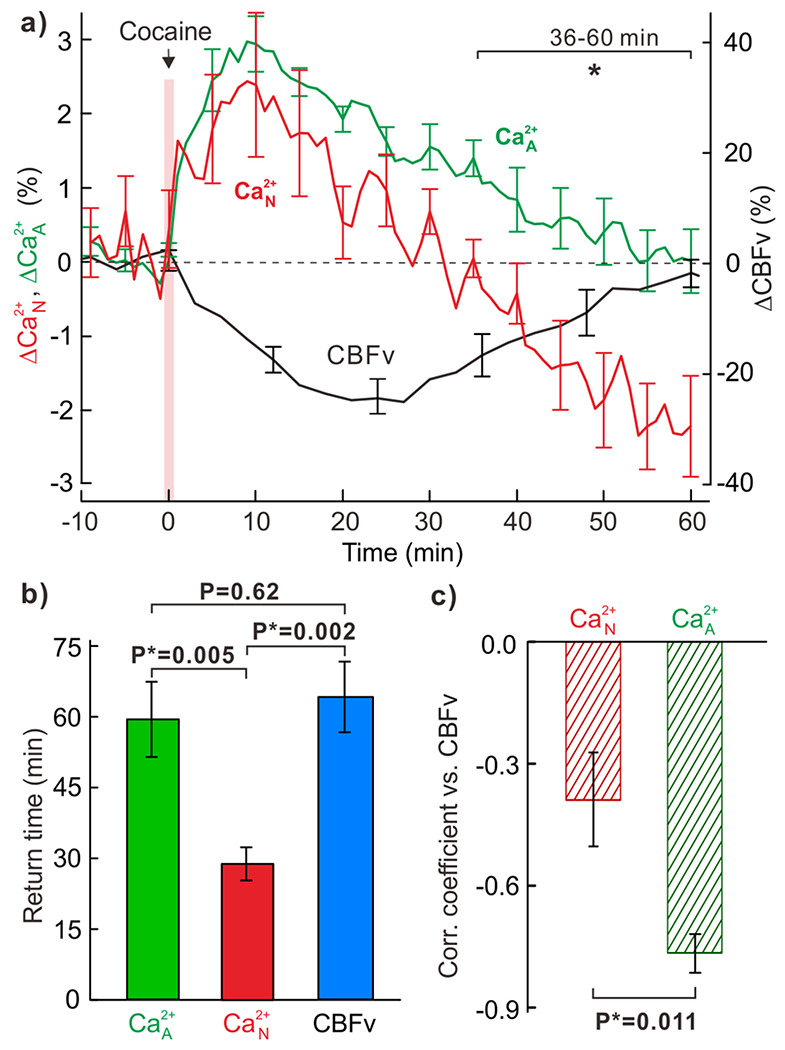
Comparisons of cocaine’s effects on neuronal Ca^2+^_N_, astrocytic Ca^2+^_A_ fluorescence and vascular CBFv in the PFC (n=7 mice), **a)** Mean ΔCa^2+^_A_ (green), ΔCa^2+^_N_ (red) and vascular ΔCBFv (black) responses to cocaine (1mg/kg, i.v.). **b)** Comparisons of return time to baseline between ΔCa^2+^_A_, ΔCa^2+^_N_ and ΔCBFv, showing that the Ca^2+^_A_ and CBFv responses to cocaine lasted significantly longer than that of Ca^2+^_N_, while there was no difference between ΔCa^2+^_A_ and ΔCBFv (p=0.62, n=7). **c)** Temporal correlations of Ca^2+^_A_ and Ca^2+^_N_ transient changes vs CBFv changes in response to cocaine.

Figure 2 illustrates the increases in Ca^2+^_N_ and Ca^2+^_A_ and the decreases in CBFv triggered by cocaine in the mouse PFC. Unlike the short-lasting dynamic change in Ca^2+^_N_ fluorescence (e.g., <30min), cocaine induced long-lasting changes in Ca^2+^_A_ and CBFv (e.g., 50-58min). To analyze the temporal relationship among these cellular and vascular changes, we studied 6 additional mice to examine the effects of acute cocaine (1mg/kg, i.v.) on neuronal Ca^2+^_N_, astrocytic Ca^2+^_A_ and microvascular CBFv dynamic responses in the PFC. Figure 3 summarizes the cocaine-induced mean changes in Ca^2+^_N_, Ca^2+^_A_ and CBFv within different vascular compartments (e.g., arteries, veins and capillaries) acquired from seven animals. The mean time-course changes in Fig. 3a indicate that cocaine induced a **Δ**Ca^2+^_N_ increase of 2.46±0.88% at t_p–N_=8.2±2.1min followed by recovery to baseline at t_r–N_=28.8±3.5min. Similarly, **Δ**Ca^2+^_A_ increased to 2.97±0.43% at t_p–A_=12.1±2.2min, but the effect was long-lasting and did not return to baseline until t_r–A_=59.5±8.0min. In parallel cocaine decreased mean vascular **Δ**CBFv to −25.1±4.9% at t_p–V_=21.0±2.9min, which slowly recovered to baseline at t_r–V_=64.0±7.5min, with a similar duration to that of **Δ**Ca^2+^_A_. Statistical comparisons of the response duration to cocaine between **Δ**Ca^2+^_N_, **Δ**Ca^2+^_A_ and **Δ**CBFv are summarized in Fig. 3b, which indicates that the response duration of Ca^2+^_A_ and CBFv to cocaine was significantly longer (~2 folds) than that of Ca^2+^_N_ (P*=0,005 and P*=0.002, respectively, n=7). No significant difference was found between t_r–A_ and t_r–V_ (P=0.62, n=7). The temporal correlations between cocaine-induced **Δ**Ca^2+^_A_(t), **Δ**Ca^2+^_N_(t) and **Δ**CBFv(t), were computed and the data derived from all 7 mice is illustrated in Supplemental **Fig.S2**. The results in Fig. 3c show a strong correlation between **Δ**Ca^2+^_A_ and **Δ**CBFv (0.765±0.048, n=7) that was significantly higher than the significant but weaker correlation between **Δ**Ca^2+^_N_ and **Δ**CBFv (0.389±0.115, P*=0.011, n=7).

### 4. Activation of GFAP-DREADDs(Gi) inhibited astrocytic Ca^2+^_A_and induced vasodilation and increased CBFv during baseline

**Figure 4:**
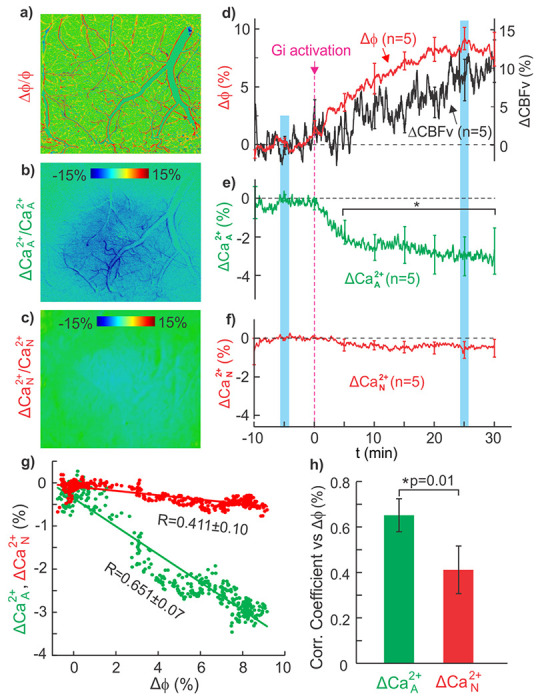
**a-c)** Ratio images of cerebrovessels and of Ca^2+^_A_ and Ca^2+^_N_ fluorescence at t=25min after GFAP-DREADDS(Gi) activation via clozapine vs their baselines at t=−5min, indicating vasodilation (red edges in **a**) and Ca^2+^_A_ fluorescence decrease (blue area in **b**) but no effects on Ca^2+^_N_ (in **c**) in PFC; **d-f**) Mean time courses of Df increase or vasodilation and DCBFv increase in **d**) and Ca^2+^_A_ decrease in **e**) but no Ca^2+^_N_ change in **f**) after DREADDS(Gi) activation (n=5 mice). **g**) Correlation analyses of ΔCa^2+^_A_(t) and ΔCa^2+^_N_(t) vs. ΔΦ(t); **h**) A comparison of correlation coefficients of ΔCa^2+^_A_(t) vs ΔΦ(t) (green bar) and ΔCa^2+^_N_(t) vs ΔΦ(t) (red bar), showing a significant difference (*p=0.01).

DREADDS is a chemogenic approach that enables subtype selective activation (Gq) or silencing (Gi) of cellular signaling (e.g., astrocytes) via clozapine activation^25^. A cocktail of two viruses consisting of 0.4μl AAV5.CAG.Flex.GCaMP6f.WPRE.SV40 and 0.4μl AAV5.GFAP.hM4D(Gi)- mCherry was injected into the PFC of GFAP-cre mice. Figure 4 shows *in vivo* results using Gi-coupled DREADDS(hM4Di) expression in astrocytes (referred as GFAP-DREADDS(Gi)) into the mouse PFC. After 4-6wks from injection, time-lapse images of Ca^2+^_A_ fluorescence and cerebrovascular networks in the PFC were continuously acquired before and after clozapine injection (0.8mg/kg, *i.p.*) at t=0min for over 40min. Clozapine instead of clozapine-N-oxide (CNO) was used to activate DREADDS(Gi) because a recent study showed that clozapine (the CNO metabolite) rather than CNO itself stimulates the DREADDS receptor^25^. Figure 4b is a representative ratio ΔCa^2+^_A_ image of a mouse PFC post clozapine (t=25min) over its baseline (t=−5min), showing Ca^2+^_A_ decreases within the blue region of brain tissue along with vasodilation (red tracks in vascular trees, Fig. 4a). Details of vascular responses to clozapine activation of DREADDS(Gi) can be visualized in Supplemental movie **VS1** and ratio image shown in Supplemental **Fig.S3**. Figure 4d summarizes the mean changes in vascular diameters and CBFv as a function of time post clozapine administration across animals (m=5 ROI/parameter/animal, n=5 mice). DREADD(Gi) activation resulted in vasodilation, with an increase of Δφ=9.17%±0.96% in mean vessel diameters (black trace) from baseline (t=−5min, Δφ=0.22%±0.35%) and an increase in ΔCBFv of 10.51 %±2.95% at 25min post clozapine compared to baseline (t=−5min, ΔCBFv=0.21%±1.18%). The time course of ΔCa^2+^_A_ in response to astrocyte inhibition is shown in Fig. 4e, revealing a significant decrease after 5min following clozapine injection (p<0.05); at t=25min post clozapine, ΔCa^2+^_A_ decreased −3.0%±1.0% over its baseline (t=−5min, ΔCa^2+^_A_=0.02%±0.16%). These results provide evidence that astrocytic signaling modulates vascular tone at baseline, thus adjusting CBFv within the brain.

To evaluate whether astrocytic inhibition would affect neuronal activity at baseline and in response to cocaine (see subheading below), we injected a mixture of viruses to express GCaMP6f into neurons (0.4ul AAV5.Syn.GCaMP6f.WPRE.SV40) and to express GFAP-DREADDs(Gi) (0.4μl AAV5.GFAP.hM4D(Gi)-mCherry) into astrocytes in the PFC of mice (n=5). Figure 4c shows a representative ratio image of neuronal ΔCa^2+^_N_ fluorescence of a mouse PFC at t=25min after GFAP-DREADDs(Gi) activation by clozapine over the baseline (t=−5min), exhibiting no neuronal Ca^2+^_N_ fluorescence changes. Figure 4f shows the mean ΔCa^2+^_N_ time courses across animals, indicating no significant changes before (−0.08%±0.06%, t=−5min) and after (−0.64%±0.43%, t=25min) GFAP-DREADDs(Gi) activation (p>0.05, m=5/animal, n=5). This result indicates that clozapine activation of GFAP-DREADDs(Gi) to inhibit astrocyte activity (Ca^2+^_A_) did not influence neuronal activity (Ca^2+^_N_) at baseline. The specificity of the DREADDs(Gi) delivery into astrocytes was corroborated with immunostained brain sections that indicated AAV5.GFAP.hM4D(Gi)-mCherry expression uniquely in astrocytes (Supplemental **Fig.S4**).

Figure 4g plots the temporal correlations between **Δ**Ca^2+^_A_(t) and **Δ**Ca^2+^_N_(t) with **Δ**φ(t), and Fig. 4h indicates that the cross-correlation between **Δ**Ca^2+^_A_ and **Δ**φ (r=0.651±0.07) was significantly higher than that between **Δ**Ca^2+^_N_ and **Δ**φ (r=0.411±0.10, P*=0.01). A similar correlation analysis between **Δ**Ca^2+^_A_(t) and **Δ**Ca^2+^_N_(t) with **Δ**CBFv(t) shows that the correlation between **Δ**Ca^2+^_A_ and **Δ**CBFv (r=0.535±0.03) was significantly higher than that between **Δ**Ca^2+^_N_ and **Δ**CBFv (r=0.347±0.05, P*=0.008) (Supplemental **Fig.S5**). These results indicate that inhibition of astrocytes resulted in vasodilation and CBFv increases, demonstrating the modulatory role of astrocytes in setting baseline vascular tone and blood flow in the brain. Though the correlations between decreases in neuronal activity and vasodilation and CBFv increases were also significant, the effect was significantly smaller.

### 5. GFAP-DREADD (Gi) inhibition of astrocytic Ca^2+^_A_ activation attenuated the decreases in CBFv and the neuronal activation triggered by acute cocaine

To examine whether inhibition of astrocytic signaling (Ca^2+^_A_) could block the CBFv decrease due to cocaine’s vasoconstricting effects, we imaged the vascular responses to cocaine in the PFC without and with GFAP-DREADDS(Gi) activation by clozapine. Animal preparations were as described in the experiment shown in Fig. 4 but extended to assess the effects of acute cocaine. For this experiment, two sets of imaging sessions were conducted with at least 2hr separation between two sequential cocaine injections: images were acquired from 10min prior (baseline) to 60min after the first cocaine injection (1mg/kg, i.v.); this was followed by a>40min no intervention period after which a clozapine injection (0.1mg/kg, 0.16ml) was given and 30min later the second *in vivo* imaging session was initiated and included a 10min baseline prior to and 60min following a second cocaine injection (1mg/kg, iv). Astrocytic Ca^2+^_A_ fluorescence and CBFv images were continuously recorded prior to and following each cocaine injection (n=3). In an additional group of animals (n=4) we assessed whether inhibition of astrocytes influenced the neuronal response to cocaine by imaging neuronal Ca^2+^_N_ and astrocytic Ca^2^ fluorescence and CBFv changes in response to cocaine without and with GFAP-DREADD(Gi) activation by clozapine.

**Figure 5:**
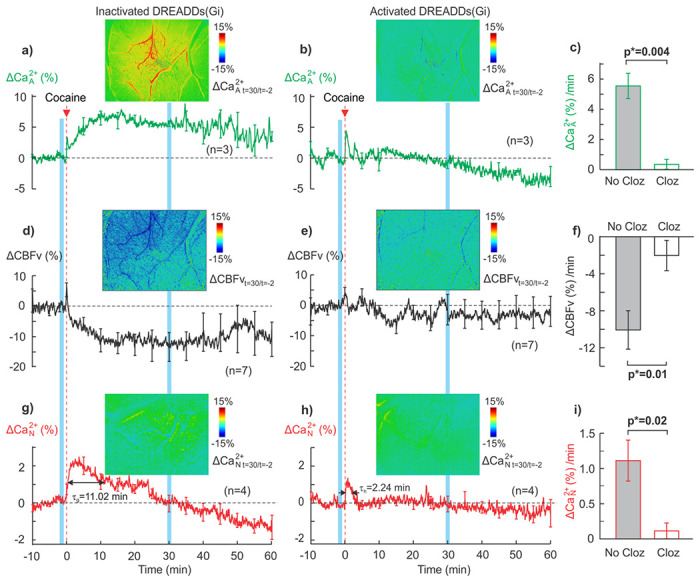
Inhibiting Ca^2+^_A_ with GFAP-DREADD (Gi) blocked cocaine-induced vasoconstriction and CBFv reduction and blunted the neuronal activation in response to cocaine in PFC. **a-b**) Cocaine-induced mean astrocytic Ca^2+^_A_ changes with time before and after GFAP-DREADD(Gi) activation (n=3), showing that GFAP-DREADD(Gi) activation inhibited cocaine-induced Ca^2+^_A_ increase. Insets: representative ratio images of Ca^2+^_A_ fluorescence at=30min after cocaine vs the baseline (t=−2min) in one animal (ΔCa^2+^_A t=30/t=2_) illustrating the blockade of cocaine-elicited ΔCa^2+^_A_ increase (a) after GFAP-DREADD(Gi) activation (**b**). **c)** Comparison of cocaine-induced mean ΔCa^2+^_A_ rates within t=0-30min, showing blockade of cocaine’s effects on ΔCa^2+^_A_ after inhibition of Ca^2+^_A_ with GFAP-DREADD (Gi) (p=0.004). **d-e**) Cocaine-induced mean CBFv changes before and after Ca^2+^_A_ inhibition by GFAP-DREADD(Gi) (n=7), showing that GFAP-DREADD(Gi) activation inhibited cocaine-induced CBFv decreases. Insets: representative ratio images of CBFv at t=30min after cocaine vs the baseline (t=−2min) in one animal (ΔCBFv_t=30/t=2_), illustrating the blockade of cocaine-elicited CBFv decreases (**d**) after GFAP-DREADD(Gi) activation (**e**). **f**) Comparison of cocaine-induced mean ΔCBFv rates within t=0–30min without and with GFAP-DREADD(Gi) activation, showing significant decreases in cocaine’s effects on ΔCBFv after inhibiting Ca^2+^_A_ with GFAP-DREADD (Gi) (p=0.01). **g-h**) Cocaine-induced mean neuronal Ca^2+^_N_ changes with time before and after Ca^2+^_A_ inhibition by GFAP-DREADD(Gi) (n=4), showing that GFAP-DREADD(Gi) activation blunted cocaine-induced Ca^2+^_N_ increases. Insets: representative ratio images of Ca^2+^_N_ fluorescence at=30min after cocaine vs the baseline (t=−2min) in one animal (ΔCa^2+^_N t=30/t=2_), showing that cocaine-induced neuronal ΔCa^2+^_N_ return to baseline at t=30min post cocaine (**g**) was shortened to 10min with astrocytes’ inhibition (**h**). **i**) Comparison of cocaine-induced mean ΔCa^2+^_N_ rates within t=0-30min without and with GFAP-DREADD(Gi) activation, showing significant decrease in cocaine’s effects after inhibiting Ca^2+^_A_ with GFAP-DREADD (Gi) (p=0.02).

Figure 5(a–b) show the temporal responses of mean astrocytic Ca^2+^_A_ to cocaine before and after DREADD(Gi) activation, in which the insets illustrate the corresponding representative ratio images of Ca^2+^_A_ fluorescence at t=30min after cocaine vs baseline (t=−2min), indicating that cocaine-induced mean astrocytic Ca^2+^_A_ increase (ΔCa^2+^_A_ (**a**) was inhibited after GFAP-DREADD(Gi) activation (**b**)). Multiple ROIs (m=5) within the PFC from each animal (n=3) were selected to track temporal Ca^2+^_A_ fluorescence changes (ΔCa^2+^_A_/Ca^2+^_A_) after cocaine and the data averaged to derive mean ΔCa^2+^_A_(t) curves in Fig. 5(a,b). For statistical comparison, the ΔCa^2+^_A_ rate, defined as the averaged per minute Ca^2+^_A_ increase post cocaine during t=0-30min was blocked from 5.55%±0.83%/min to 0.35%±0.32%/min (p*=0.004) after GFAP-DREADD(Gi) activation (Fig. 5c), consistent with inhibition of cocaine-induced Ca^2+^_A_ increase in astrocytes. Similarly, Fig. 5(d–e) show the mean temporal CBFv responses (ΔCBFv/CBFv) to cocaine before and after GFAP-DREADD(Gi) activation (n=7, including animals both with astrocytic-GCaMP6f or neuronal-GCaMP6f and GFAP-DREADD(Gi) expressions) as well as their corresponding representative ratio images to illustrate the blunting of cocaine-induced ΔCBFv decrease. The CBFv decrease from −0.72%±1.42% at baseline (t=−2min) to −14.64%±4.75% at t=30min after cocaine (Fig. 5d) was reduced to −1.43%±5.02% (t=30min) from its baseline −0.47%±1.44% (t=−2min) with GFAP-DREADD(Gi) activation (Fig. 5e). Figure 5(f) indicates that cocaine-induced ΔCBFv decrease was reduced significantly from −10.1%±2.1%/min to −2.03%±1,6%/min after GFAP-DREADD (Gi) activation (p*=0.01, n=7). Demonstration of cocaine-induced vascular response without and with astrocytes’ inhibition via DREADDs(Gi) activation can be visualized in Supplemental movies **VS2** and **VS3**, respectively. It showed that cocaine induced vasoconstriction, but this effect was eliminated by astrocytes’ inhibition after DREADDs(Gi) activation (Supplemental **Fig.S6**). These results support our hypothesis that blocking Ca^2+^_A_ increases would abolish cocaine-induced vasoconstriction and prevent CBFv decreases.

While GFAP-DREADD(Gi) targets astrocytes and inhibits cocaine-induced Ca^2+^_A_ increase (Fig. 5a), it was unclear whether it could indirectly modify neuronal responses to cocaine. Figure 5(g–h) shows the temporal responses of mean neuronal Ca^2+^_N_ to cocaine before and after DREADD(Gi) activation to inhibit Ca^2+^_A_. Unlike the long-lasting response of astrocytes to cocaine (>60min shown in Fig. 5a) the neuronal Ca^2+^_N_ increase returned to baseline at 30min after cocaine as illustrated in Fig. 5g. The full-width-half-maximum duration of cocaine-induced Ca^2+^_N_ increase was reduced from **τ_g_**=11,02±1.85min to **τ_h_**=2.24±0.62min after DREADD(Gi) activation. The ΔCa^2+^_N_ rate decreased from 1.11%±0.29%/min to 0.11%±0.1%/min after GFAP-DREADD(Gi) activation (Fig. 5i), thus indicating a significant reduction of cocaine-induced neuronal activation (p*=0.02, n=4). Taking together with the effect of DREADDS(Gi)’s blockade on cocaine-induced CBFv decreases (Fig. 5f), this result indicates that cocaine’s effects on CBFv and neuronal activity (Ca^2+^_N_) were modulated by Ca^2+^_A_ signaling. Thus, inhibition of astrocytic activity might help alleviate cocaine associated PFC dysfunction resulting from improper tissue perfusion and the decrease in neuronal reactivity might help reduce compulsive drug taking,

## Discussion

Neuroimaging has advanced our understanding of the brain but there is need for tools with cellular and capillary resolutions capable of distinguishing signaling from distinct cell types alongside the dynamics of the vascular responses in order to investigate the roles of astrocytes and neurons on cocaine’s effects in the neuro-glio-vascular (NGV) circuit. Here we integrate fluorescence imaging and ultrahigh-resolution ODT to form multi-channel fluorescence and optical coherence Doppler microscopy (fl-ODM), and apply it to study cocaine effects on the NGV circuit within mouse PFC. We explored how acute cocaine affects astrocytic Ca^2+^_A_ and neuronal Ca^2+^_N_ activities and CBFv, and tested the hypothesis that cocaine induces Ca^2+^_A_ accumulation resulting in vasoconstriction and CBFv decreases that disrupt neurovascular coupling. We then investigated the role of Ca^2+^_A_ in cocaine elicited vasoconstriction, CBFv decrease and Ca^2+^_N_ change, and tested the hypothesis that reducing Ca^2+^_A_ via DREADDS(Gi) could relieve acute cocaine induced vasoconstriction, CBFv decreases and Ca^2+^_N_ increases.

### 1) Cocaine-induced Ca^2+^_A_ accumulation resulted in vasoconstriction and CBF decreases that disrupted neurovascular coupling

Astrocytic Ca^2+^_A_ signaling cascades are involved in the communication between neurons and astrocytes, and astrocyte-to-astrocyte communicate via Ca^2+^ waves that propagate signaling over a large range^3^. Ca^2+^_A_ may also regulate CBF independently of synaptic activity^26^ and activation of Ca^2+^_A_ may release glutamate to regulate synaptic homeostasis^5^. These findings highlight the role of astrocytes in modulating neuronal function and hemodynamics.

Prior studies from our group and others have shown that astrocytes contribute to vasodilation during neurovascular coupling and to vasoconstriction that subsequently restores vascular tone ^12,27^. However, studies on the contribution of astrocytes to cocaine’s effects on the brain have mostly focused on its synaptic and circuitry regulation associated with its rewarding and addictive effects^28^. Only few studies have investigated the effects of acute cocaine on astrocyte activity, including an *in vitro* study that used brain slices from the nucleus accumbens incubated with cocaine that reported increases in Ca^2+^ transients in astrocytes^29^. To our knowledge, there is no *in vivo* study that has simultaneously imaged Ca^2+^_N_ and Ca^2+^_A_ fluorescence alongside CBFv changes in response to cocaine, thus the hybrid fl-ODM imaging platform reported here uniquely enables us to characterize how astrocyte and neuronal networks in the PFC interact and mediate the associated local neurovascular responses to cocaine.

Cocaine directly affects both cerebral blood vessels and neuronal activity in the brain^30–33^. Glia, in particular astrocytes, are involved in neurovascular coupling, which modulates hemodynamics in response to changes in neuronal activity^34^. Neurovascular coupling can be disrupted by use of addictive drugs such as cocaine and by disease processes such as Alzheimer’s disease and other dementias. The ability to distinguish neuronal from vascular effects remains a challenge, partially due to technical limitations of neuroimaging techniques to differentiate vascular from neuronal and glial effects at high spatiotemporal resolutions. Here, we applied fl-ODM to study cocaine’s effects on the neurovascular network and on neuronal and astrocytic activities in the PFC. Specifically, we simultaneously imaged activations of Ca^2+^_N_, Ca^2+^_A_ fluorescence and local CBFv changes elicited by an acute cocaine challenge (1mg/kg, *i.v*). Findings revealed a temporal association between cocaine-induced CBFv decreases (due to vasoconstriction) and the long-lasting astrocytic activation. Analysis of temporal correlations showed that cocaine-induced Ca^2+^_A_ increases had strong negative correlations with the CBFv decreases. These findings are in agreement with our recent results obtained in separate groups of animals^35^. In that study we used single virus containing GCaMP6f (not jRGECO1a) delivered into the somatosensory cortex and reported that cocaine-induced neuronal Ca^2+^_N_ increases recovered by 30min followed by a downshoot similar to our observations in this study but in the PFC (Fig. 3a above). In our previous study we also showed that cocaine-induced Ca^2+^_N_ changes were inversely correlated with temporal changes in tissue oxygenation^35^. The duration of neuronal activation in response to cocaine observed here and in our prior study is consistent with the pharmacokinetics of intravenous cocaine in the brain^36–38^ and to the duration of striatal dopamine increases^39^. The inverse association between Ca^2+^_N_ and tissue oxygenation indicates that cocaine’s effects on neuronal activation and deactivation underly the changes in tissue oxygenation and might also underlie the reductions in brain glucose metabolism reported during cocaine withdrawal^40,41^. Although we had observed that the lasting increases in Ca^2+^_A_ induced by cocaine were associated with vasoconstriction as assessed via quantification of vessel diameter changes^35^, the measurements of Ca^2+^_A_ and Ca^2+^_N_ changes were done in separate groups of mice, whereas in the current study the fl-ODM enabled us to assess the dynamic Ca^2+^_A_ and Ca^2+^_N_ responses simultaneously in the same animals. This was crucial for assessing the role of astrocytes in mediating the neuronal responses to cocaine alongside vascular function. It also allowed us to control for group variability. For example, in our prior study using separate groups of mice, the amplitude of cocaine-induced Ca^2+^_A_ was 3 fold lower than that of the Ca^2+^_N_ response^35^, whereas in the current study that measured them in the same animals there were no differences between the peak values of Ca^2+^_A_ and Ca^2+^_N_ (i.e., 2.97±0.42% vs 2.46±0.42%, p=0.82). This discrepancy likely reflects differences between the groups (i.e., WT vs GFAP-cre mice) in our prior study. Nevertheless, this study extended our previous findings and showed for the first time that inhibition of Ca^2+^_A_ also attenuated cocaine-induced Ca^2+^_N_ increases.

### 2) Reducing Ca^2+^_A_ via DREADDS(Gi) relieved cocaine-induced vasoconstriction, CBFv decreases and Ca^2+^_A_ increases

At baseline the inhibition of astrocytic activation decreased Ca^2+^_A_ and resulted in vasodilation and CBFv increases but did not affect neurons in PFC. This suggests that at baseline astrocytes, but not neurons, mediate vascular tone, which is consistent with previous reports with two-photon microscopy (Takano et al, 2006). During a cocaine challenge, the inhibition of astrocytic activation prevented the CBFv decreases triggered by cocaine-induced vasoconstriction. Different from baseline, inhibition of astrocyte activation also blunted neuronal activation by cocaine. Together, these findings indicate the involvement of astrocytes in mediating cocaine’s effects on vasoconstriction and in modulating the neuronal responses to cocaine. Though the mechanisms by which astrocytes affect neuronal responses to cocaine are unclear it is possible that it could involve astrocytes’ role in terminating the action of glutamate released by neurons via the glutamate-glutamine cycle^3^ and in restoring synaptic glutamate homeostasis after cocaine exposure^5,24^. Moreover, manipulating astrocyte function attenuate relapse after cocaine withdrawal^5,42,43^.

Human studies have reported neuronal, astrocytic and vascular pathology in the brain of individuals with cocaine use disorder (as well as other drugs of abuse) that encompassed neuronal loss, reduction of glial fibrillary acidic protein (GFAP)-immunopositive astrocytes and reactive and degenerative changes of cerebral microvessels^4^. These observations imply that drugs including cocaine initiate a cascade of interacting toxic processes in the NGV circuit that are likely to contribute to the cognitive and behavioral changes observed in drug users.Intracellular Ca^2+^ increases are associated with cell death^3^; thus the cocaine-induced cellular Ca^2+^ increases that we observed are clinically relevant, particularly since they occur in parallel with CBF decreases and hypoxia^40^. The use of a new fl-ODM enabled us to separate cocaine’s effects on astrocytes, neurons and vascular networks to underpin their contributions to PFC dysfunction induced by cocaine. We focused on the PFC since clinical studies provide ample evidence of PFC dysfunction in drug users^1,31^ that is implicated on the loss of control over drug taking^44^. Relevant to our findings is a recent report that mediation of Ca^2+^_A_ signaling ameliorated neuronal death and reduced behavioral deficits after ischemic stroke^45^. We had also reported that nifedipine (Ca^2+^ antagonist and vasodilator^46^) prevented cocaine-induced CBF decreases and neuronal Ca^2+^ increases in PFC and reduced cocaine intake. In that study we interpreted nifedipine’s actions to indicate neuronal effects, our current findings suggest that blockade of L-type Ca^2+^ channels in astrocytes are likely to be involved^47,48,49,50^. Our current findings provide new insights into cocaine’s effects on the NGV interactions that may provide new targets for development of novel addiction treatments.

A limitation for our study was that experiments were conducted in anesthetized mice using isoflurane to avoid artifacts from animal motion during imaging. Isoflurane-induced vasodilation might have facilitated the detection of cocaine-induced vasoconstriction, especially in capillaries and its anesthetic effects might have attenuated the sensitivity of neurons^52^ and perhaps also of astrocytes to cocaine. Another limitation was that in order to compare cocaine’s effects with and without blockade of astrocytic activation by DREADDs(Gi), each animal was administered cocaine twice, which might have resulted in tolerance. In our study we gave the second cocaine dose 2hrs after the first one, for we had previously shown that the hemodynamic responses to cocaine in PFC between two cocaine doses separated by a 2hr interval did not differ from one another^51^. Nonetheless, we cannot completely rule out the potential effects of tolerance to the second cocaine dose. Another limitation was the viral delivery protocol, which only allowed delivery of two types of viruses into the PFC to minimize risk of animal losses from viral injections and to ensure sufficient amount of viruses (e.g., 0.4ul per virus) to be expressed in each cell type (e.g., neuron, astrocytes) for fluorescence detection. The complexity of the studies also restricted the sample sizes of animals investigated, which precluded us to compare male and female responses to cocaine. Finally, the mechanisms by which astrocytes mediate vasoconstriction or neuronal responses to cocaine are unclear.

In conclusion, we observed that at baseline astrocytes mediated vascular tone but did not change neuronal activity whereas during a cocaine challenge they not only prevented the CBFv decreases but also attenuated cocaine-induced Ca^2+^_N_ increases in PFC, Our findings provide further evidence for the role of astrocytes in modulating NGV interactions in responses to cocaine both via direct effects in cerebral blood vessels and indirectly via their modulation of neuronal reactivity. Strategies to inhibit astrocytic activity could be promising in addressing vascular and neuronal toxicity from cocaine misuse. In addition, our findings demonstrate the capabilities of fl-ODM for distinguishing activities of different cells (e.g., neuron, astrocytes) and its compatibility with other imaging tools for simultaneous monitoring of hemodynamics (or other processes).

## Methods

### Animals:

All experiments were carried out according to National Institutes of Health guidelines and were approved by the Institutional Animal Care and Use Committee of Stony Brook University. GFAP-cre mice, obtained from Jackson Laboratory and maintained as a heterozygous line, were used for experiments when they reached the age between postnatal 60–70 days (P60-P70). All the information regarding the generation and genotyping of this line is available at https://www.jax.org/strain/024098. The physiological conditions of the mice (Respiration, body temperature, etc.) were continuously monitored during the experiments to ensure similar physiological status for all mice.

### Viral expression of genetically encoded Ca^2+^ indicators (GECIs) in neurons and astrocytes:

To simultaneously image the fluorescence from green and red GECIs (GCaMP6f, jRGECO1a) differentially expressed in astrocytes and neurons, a mixture of viral vectors containing 1:1 AAV5.CAG.Flex.GCaMP6f.WPRE.SV40 (100835-AAV5, Addgene) and AAV1.Syn.NES-jRGECO1a.WPRE.SV40 (100854-AAV1, Addgene) was injected into the PFC (A/P: +2.5mm; M/L: 0.5mm; D/V: −0.5mm) of GFAP-cre mice for astrocytic and neuronal uptake under isoflurane anesthesia. A total volume of 0.8μl was slowly injected (e.g., ~0.1μl/min) using a Hamilton syringe, and after the microinjection the needle was left intact for additional 5-10min to avoid backflow or leakage of the injected viral vectors. To monitor the vascular and Ca^2+^_A_ responses to DREADD(Gi) activation by clozapine and examine whether Ca^2+^_A_ was involved in cocaine-induced vasoconstriction, a mixture of two viral vectors containing 0.4μl AAV5.CAG.Flex.GCaMP6f.WPRE.SV40 and 0.4μl AAV5.GFAP.hM4D(Gi)-mCherry (50479-AAV5, Addgene) was injected into the same PFC region. During viral injection, mice were anesthetized with inhalation of 2% isoflurane mixed with pure oxygen and their heads mounted on a stereotaxic frame while we monitored their physiology. After completion of the procedure, the mice were monitored daily for a few days to ensure that they fully recovered from the surgery.

### Cranial window implantation:

A region of interest on the mouse PFC (A/P: +2.5; M/L: 0.5; D/V: −0.5 mm) was selected, where the cortical bone was first thinned using a dental drill and then carefully removed, leaving the dura intact. The explored brain region was treated with dexamethasone sodium phosphate (50989-437-12, VEDCO) and then immediately covered by a 3.5×4.5mm^2^ coverslip and sealed with biocompatible glue. Dental cement was spread around the edges of the coverslip to further secure its attachment with the skull for repeated imaging.

### *In vivo* time-lapse fl-ODM imaging:

Mice were anesthetized using inhalational isoflurane (1.5%~2.5%) and the head mounted onto a stereotaxic frame. A custom fl-μODM developed in our lab was used to simultaneously image the CBFv networks and Ca^2+^_N_ and Ca^2+^_A_ fluorescence in a spectral-multiplex, time-sharing mode in the PFC (Fig. 1). For astrocytic GCaMP6f-Ca^2+^_A_ and neuronal jRGECO1a-Ca^2+^_N_ fluorescence imaging, light beams from 10ms-duration pulsed narrow-band blue LED at 488nm and yellowish-green LED at 560nm of a light engine (Aura III, Lumencor) were combined in a light guide to illuminate a modified fluorescence microscope (FN1 Nikon) for excitation. By spectral multiplexing, the epifluorescence cube C2 selectively allowed green Ca^2+^_A_ (500-540nm) and red Ca^2+^_N_ (574-670nm) emission from the mouse cortex (3·4mm^2^) to be acquired by a sCMOS camera (Zyla 5.5, Andor) in a time-sharing mode synchronized with the excitation pulses at up to 80fps. The recorded Ca^2+^_A_ and Ca^2+^_N_ activity was quantified as the relative fluorescence change (ΔF/F). For vascular imaging, a full-size 3D μODT image at 1.3um of mouse CBFv networks (2.4·2·1.2mm^3^) was acquired in ~15min, and time-lapse μODT images over a smaller volume (e.g., 2·0.3·1.2mm^3^) were acquired per 45s or less for tracking flow dynamic changes (ΔCBFv) along with Ca^2+^_A_ and Ca^2+^_N_ activations. Similarly, the flow network change was quantified as the ratio image (ΔCBFv/CBFv).

### Immunohistochemistry:

After *in vivo* imaging studies, the mouse was perfused transcardially with 0.1M PBS, followed by fixation with 4% paraformaldehyde in 0.1M PBS. The frozen brain was sliced to 40~50μm in thickness. For GCaMP6f signal enhancement to astrocytes, the antibody [chicken anti-GFP (1:200) antibody] to green fluorescence was used as the primary antibody followed by an Alexa Fluor 488 anti-chicken for GFP (1:200) conjugated secondary antibody. To identify GFAP-DREADDs(Gi) expression into astrocytes, antibody was used to enhance green fluorescence emission in astrocytes, but no immunostaining was used for GFAP-DREADDS(Gi). The jRGECO1a expression to neurons was imaged with a confocal fluorescence microscope (A1, Zeiss) without immunostaining for fluorescence enhancement.

### Statistics:

All data are presented as mean±s.e.m. Data were analyzed by one-way or two-way mixed model analysis of variance (ANOVAs) and the Holm-Sidak method was used for post-hoc analysis. Comparison made between two different groups (e.g., ΔCa^2+^_N_-Δφ vs ΔCa^2+^_A_-Δφ, cocaine-induced ΔCa^2+^_A_ without vs with DREADDS(Gi) activation) was analyzed using Student’s t-test. If the P-value is less than 0.001, it is reported as P<0.001; otherwise, precise P-values are provided for each test. All statistical tests were performed using SigmaStat software (Systat Software Inc), with alpha levels set at 0.05 to report significance.
